# Psychosocial determinants of parental human papillomavirus (HPV) vaccine decision-making for sons: Methodological challenges and initial results of a pan-Canadian longitudinal study

**DOI:** 10.1186/s12889-016-3828-9

**Published:** 2016-12-05

**Authors:** Samara Perez, Ovidiu Tatar, Gilla K. Shapiro, Eve Dubé, Gina Ogilvie, Juliet Guichon, Vladimir Gilca, Zeev Rosberger

**Affiliations:** 1 Department of Psychology, McGill University, 1205 Dr. Penfield Avenue, Montreal, QC H3A 1B1 Canada; 2 Lady Davis Institute for Medical Research, Jewish General Hospital, 4333 Cote Ste-Catherine Road Room 214, Montreal, QC H3T 1E4 Canada; 3 Institut National de Santé Publique du Québec, 2400 D’Estimauville, Quebec, G1E 7G9 Canada; 4 Faculty of Medicine, University of British Columbia, BC Women’s Hospital and Health Centre, Room H203G, 4500 Oak Street, Vancouver, BC V6H 3N1 Canada; 5 Community Health Sciences, Faculty of Medicine, University of Calgary, 3280 Hospital Drive N.W, Calgary, AB T2N 4N1 Canada; 6 Louise Granofsky-Psychosocial Oncology Program, Segal Cancer Center, Jewish General Hospital, 4333 Cote Ste-Catherine Road, Montreal, QC H3T1E4 Canada

**Keywords:** Human papillomavirus, Cancer prevention, Vaccination, Determinants of health, Health decision-making, Health behavior, Precaution Adoption Process Model, Parents, Boys, Knowledge, Attitudes, Beliefs

## Abstract

**Background:**

HPV vaccination decision-making is a complex process that is influenced by multiple psychosocial determinants. Given the change in policy recommendation to include males in routine HPV vaccination, our goals were to assess the HPV vaccination uptake in Canada, to understand *where* Canadian parents were situated in the HPV vaccine decision-making process for their son, *how* they changed over time and *which* psychosocial determinants were relevant for this process.

**Methods:**

We used an online survey methodology and collected data from a nationally representative sample of Canadian parents of boys aged 9–16 at baseline (T1, February 2014) and at 9 months’ follow-up (T2). Our analyses were guided by the Precaution Adoption Process Model (PAPM), a theoretical health behavior model that classifies parents in one of six stages: unaware, unengaged, undecided, decided not to vaccinate, decided to vaccinate and those who had already vaccinated their sons. Rigorous methods were used to filter out careless responders: response variance, bogus items, psychometric antonyms and psychometric synonyms.

**Results:**

At T1 and T2, we received 3,784 and 1,608 respectively completed questionnaires; after data cleaning 3,117 (T1) and 1,427 (T2) were retained. Less than 3% of boys were vaccinated at both time points. At both T1 and T2, most parents (over 70%) belonged to the earlier vaccination adoption stages: 57% were unaware (T1) and 15.3% (T2); 20.9% were unengaged (T1) and 32.4% (T2); and 9.1% were undecided (T1) and 25.2% (T2). At follow-up, 37.7% of participants did not move from their initial PAPM decision-making stage. Most parents (55%) preferred to receive information from their healthcare provider (HCP) but only 6% (T1) and 12% (T2) had actually spoken with a HCP about the HPV vaccine for their son.

**Conclusions:**

HPV vaccination uptake in Canadian boys was very low in the absence of a publicly funded HPV vaccination programs for boys. Optimal HPV information preferences were identified and can be used in interventions to increase HPV knowledge and increase HPV vaccine uptake. Intentions to vaccinate or planning to speak to one’s HCP did not translate into action for most parents over the 9-month follow up; this finding is critical to consider to inform implementation strategies. Methodological challenges are described and suggestions for future research are offered.

**Electronic supplementary material:**

The online version of this article (doi:10.1186/s12889-016-3828-9) contains supplementary material, which is available to authorized users.

## Background

The prevention of human papillomavirus (HPV)-associated diseases is an increasingly prominent public health issue. HPV is the most common sexually transmitted infection (STI) and accounts for 4.8% of the worldwide cancer burden [1, 2]. HPV has been traditionally viewed as an infection that impacts females [3, 4], even though it poses a significant disease burden for males. Current data suggests that 100% of cervical, 88% of anal, 70% of vaginal, 50% of penile, 43% of vulvar and 13–56% of oropharyngeal cancers are attributable to HPV [3]. Like females, males are at risk also for contracting HPV-associated genital warts (GW), which can negatively impact quality of life [3].

The quadrivalent vaccine, Gardasil® (Merck) protects against four strains of HPV: two oncogenic strains (HPV 16, 18), and two that cause GW (HPV 6 and 11) [5]. Epidemiological studies from Australia, Canada, UK and the US demonstrate population level reductions in the rates of HPV, GW, and cervical cancer lesions after introduction of HPV vaccine programs for girls [6–11]. With strong empirical evidence for both vaccine safety and efficacy [5], the HPV vaccine is an important innovation in cancer prevention [6, 12].

In 2007, in Canada, the HPV vaccine (Gardasil®) was recommended for females and subsequently rolled out for females only in school-based immunization programs [13]. As the research evidence grew, demonstrating the burden of HPV-associated diseases in males, many argued for vaccination of males [14]. Inclusion of males in HPV immunization programs grew further because: 1) HPV vaccine uptake rates among females are failing to reach sufficient levels (of at least 70%) to confer herd immunity to heterosexual males, 2) female-only programs do not offer protection to men having sex with men (MSM); and 3) a gender specific vaccine raises issues of equity [14–22].

Presently, all Canadian provinces and territories offer free, school-based HPV vaccination programs for females. Canada’s National Advisory Committee on Immunization (NACI, 2012 and 2015) recommends HPV vaccine for females and males aged 9–26 [2]; this recommendation is consistent with that of other nations (e.g., US [23] Australia [24] and some of the European Union, e.g. Germany (Saxony), Italy (Emilia-Romagna, Sicily) [25, 26]. In February 2013, Australia was the first country to extend national vaccination programs for boys. In Canada, the HPV vaccination program for males has unfolded as follows. In September 2013, Prince Edward Island (PEI) began including boys in grade 6 in their school-based HPV vaccination programs. Alberta and Nova Scotia subsequently followed in September 2014 for grade 5 boys and in autumn 2015 for grade 7 boys respectively. In September 2015, British Columbia (BC) began offering the HPV vaccine without cost for “at risk” males e.g., MSM and ‘street-involved’ youth [27]. Similarly, as of January 2016, Quebec offers the HPV vaccine without cost to MSM aged 9–26. Beginning in September 2016, Ontario, Quebec and Manitoba will include boys in their school-based programs (grades 7, 4 and 6, respectively) [14, 28]. In contrast with the female programs, only Alberta and Ontario offer catch-up programs for older boys. When this research study was developed (2012), no HPV vaccinations programs for males existed in Canada or elsewhere in the world.

The examination of the attitudinal, behavioral, cognitive, social, cultural and logistical determinants (hereafter referred to as psychosocial) that influence the HPV-vaccine decision-making is a growing area of research [29–36]. Because HPV was traditionally considered an (an infection) that affects females only, the vast majority of behavioral research has been conducted among samples of females or parents of daughters [31, 37]. To the best of our knowledge, there are very few studies examining HPV vaccine decision-making that were conducted exclusively among parents of boys [35, 37]. In the Canadian context, only two studies outside the present one examine the psychosocial decision-making process among Canadian parents of sons; both studies were conducted before the HPV vaccine was recommended for males and therefore the outcome variable reflects *intentions* to vaccinate rather than *actual* vaccination uptake [38, 39].

Further, experts in HPV vaccine behavioral research recommend using theoretical health behavior frameworks to better understand the psychosocial determinants that influence an individual’s vaccine decision-making process [30, 40, 41]. Many studies that examine the correlates of HPV vaccine decision-making utilize the Health Belief Model (HBM) [31, 42]. The linear HBM attempts to understand better HPV vaccine decision-making by focusing on attitudes and beliefs about the costs and benefits of HPV vaccination that are relevant *only* to people who have been engaged (or are presumed to be engaged) sufficiently by the HPV vaccination to have formed such beliefs [43]. As such, most existing studies examine the psychosocial determinants that predict vaccination intentions and/or uptake are for a group of individuals who are assumed to be already aware and engaged in HPV vaccination [31, 37, 42]. Since this group does not include everyone—and with respect to HPV vaccination likely captures *few* parents because HPV vaccination for males is relatively new and many parents may not yet have formed their beliefs—there are likely other stages in adopting HPV vaccination. The Precaution Adoption Process Model (PAPM) is a categorical stage theory, which aims to identify *all* the stages involved when people commence health-protective behaviors. The PAPM is therefore appropriate to apply to parental decision-making about HPV vaccination to determine the psychosocial determinates that lead parents to move from one stage to the next, and ultimately to vaccinate their child [44].

The PAPM consists of *six* 1 distinct stages of health decision-making: 1) *unaware* of the health behavior); 2) *unengaged* in the decision; 3) *undecided*; 4) *decided not to* act; 5) decided to act (intending); and 6) acting (vaccinated). As opposed to linear models, the PAPM staged model acknowledges the fact that transition between stages can be explained by different psychosocial determinants, i.e. there are differences between determinants which influence the transition from stage 1 to 2 compared to the determinants which influence the transition from stage 5 to 6.

Using a longitudinal design and online survey methodology guided by the PAPM, we surveyed a national sample of Canadian parents of boys to understand *where* Canadian parents currently stand in the HPV vaccine decision-making process for their son and w*hich* psychosocial determinants influence their HPV vaccination decision-making process. Importantly, the present study was conducted just before several Canadian provinces began to include males in their school-based public vaccination program. This created a unique opportunity to provide baseline data about HPV vaccine uptake in the absence of publicly funded programs, and to evaluate the impact of recent recommendations of male HPV vaccination.

The study objectives were:

1. To provide an estimate of HPV vaccine uptake among males in Canada;
2. To classify *where* Canadian parents’ stand in the HPV vaccine decision-making process for their son (s) using the PAPM, at baseline (Time 1, T1) and at follow-up 9 months later (Time 2, T2);
3. To describe *how* Canadian parents’ changed in their HPV vaccine decision-making process from T1 to follow-up (T2); and
4. To describe and analyze *which* psychosocial determinants influence parents’ HPV vaccine decision-making process i.e., PAPM stage

This research article will present a comprehensive description of the study methodology, sample characteristics as well as the results for objectives 1–3. Descriptive result for objective four, specifically for the following psychosocial determinants: HPV and HPV vaccine Knowledge, HPV vaccination information sources, health behaviors (i.e., primary decision-maker, routine check-ups with a healthcare provider (HCP), and childhood immunization practices) and implementation intentions will be presented. A more comprehensive statistical analysis of the psychosocial determinants of PAPM stages over time i.e., objective 4, is under way.

## Methods

### Recruitment

**Fig. 1:**
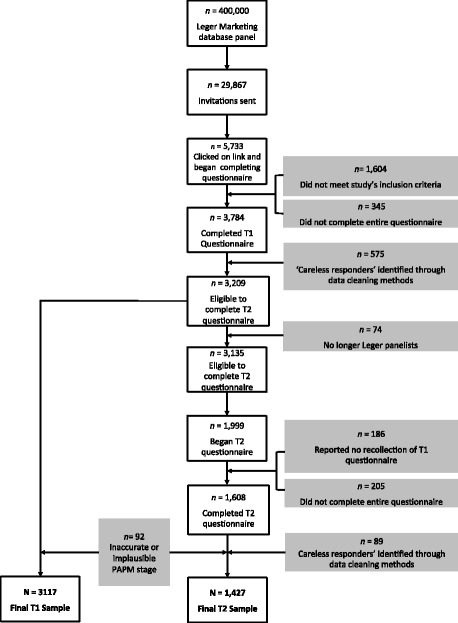
Flow diagram of study participants

The population of interest was Canadian parents and/or guardians (hereafter referred to as parents) of 9–16 year-old boys. We selected this population because it covers the current NACI HPV vaccine recommendation for males aged 9–26, and because after the age of 16, virtually all Canadian minors may make a vaccination choice without parental consent [45, 46]. Data collection was facilitated by Leger^2^ a polling and market research firm that maintains a national panel of 400,000 Canadians across the 10 provinces. The first wave of data collection occurred between February 7 and 25, 2014. E-mail invitations to complete a ~20-min online questionnaire were sent to 29,867 panelists who met the study’s inclusion criteria (i.e., those who had a 9–16-year-old son living in their household) according to data Leger maintains on their panelists. These invitations were followed by 16,004 reminder emails (between 1 and 3 emails per participant). The second wave of data collection occurred between October 27 and November 19, 2014. E-mail invitations were sent to 3,135 participants who were eligible from T1 to participate at T2. These invitations were followed by 8,341 reminder emails were (between 1 and 5 emails). At the time of data collection, HPV vaccination for males in Canada was just commencing; PEI had initiated a school-based HPV vaccination program for boys five months before (~Sept 2013) our T1 data collection, while Alberta followed one month ahead of (~Sept 2014) our T2 data collection. See Fig. 1 for a detailed schematic of study participants following study recruitment and data cleaning.

### Procedure

Prior to beginning the questionnaire, participants were asked first if they prefer to answer the questionnaire in English or French and were provided the questionnaire in the language of preference. Participants were also asked to agree to answer the questions truthfully and thoughtfully or were excluded from completing the study. Participants were also asked at the beginning of the questionnaire to provide a name, nickname, initials or abbreviations for their son who is between the ages of 9 and 16 and who has had the nearest birthday. Using intelligent programming, the provided sons’ initials, name or nickname (e.g., *Dan*) was then replaced for *my son* in most items, making the questionnaire individualized for each participant. In this way, participants were asked about their beliefs and attitudes relative to one specific son. Participants were informed that their son’s name will not be used in any other way by the researchers. Participants were required to complete every item, obviating the problem of missing data. For 16 sensitive questions, “I prefer not to answer” was a response option provided. In order to further ensure recollection of answering the questionnaire at T1, participants were asked at T2 “Do you remember completing the survey related to the HPV vaccine about *my son*?” Participants who indicated no recollection were not invited to participate at T2. Respondents were compensated 3$ per completed questionnaire at both T1 and T2.

### Measures

#### Questionnaire development

A 2010 systematic review of measures used in HPV vaccine acceptability research called for standardized theoretical and operational definitions of constructs [33]. This recommendation included: 1) utilizing theory to guide construct definitions [47, 48]; 2) employing cognitive testing [49]; 3) reviewing measures by panels of experts [39, 47, 48, 50]; 4) measuring intentions and actual behavior through clear definitions (e.g., asking about compliance to recommended number of doses) [51]; and, 5) development of constructs to take into account language and literacy levels [48, 49, 52]. Our questionnaire development adhered to all five recommendations, including a ‘think aloud’ pilot testing of the questionnaire with 20 parents of 9–16-year-old boys. The questionnaire was developed, reviewed and approved by a bilingual panel of seven experienced HPV researchers.

The English questionnaire was translated into French by a specialized translation firm and reviewed for accuracy by an independent bilingual group of professionals (*n* = 5) working in the healthcare field. The questionnaires were virtually identical at T1 and T2 except for minor differences (e.g., demographic items were removed at T2; items related to conspiracy beliefs were added at T2). The complete questionnaire^3^ contained 165 items at T1 and 191 items at T2, and is available by contacting the corresponding author. A summary of the questionnaire constructs, sample items and response options are provided in Additional file 1.

### Socio-demographics (12 items)

The first 12 items were standard socio-demographic variables (e.g., province, education, religion) selected from Statistics Canada 2011 Census questionnaire. We compared our sample to data the authors requested from Statistics Canada’s National Household Survey (2011) of participants who met our inclusion criteria i.e., parents of 9–16-year-old sons (*n* = 2,336,115) residing in the 10 Canadian provinces in order to assure the generalizability of our results to the entire Canadian population. First, chi-square tests were performed to examine if there were any significant differences in socio-demographics between our T1 and T2 samples. Next, chi-square tests were performed to examine if there were any significant differences in socio-demographics between T1 and Statistics Canada’s sample (see Table 1). Because statistical significant differences (*p* < 0.05) in proportions do not indicate the size of the difference, we further calculated Cohen’s h [53] (see Table 1). Consistent with Cohen’s recommendations, we interpreted h ≤ 0.2 as small, h = 0.5 as medium and h ≥ 0.8 as a large difference [53].

**Table 1:** Socio-demographics for T1, T2 and Statistics Canada national household survey samples. Test of proportions and effect size between T1 to T2 and T1 to Statistics Canada sample

	Time 1 *n* = 3117		Time 2 *n* = 1427		StatCan *n* = 2336115		T1:T2	Effect size Cohen’s h	T1: StatCan	Effect size Cohen’s h
	*n*	%	n	%	*n*	%	χ^2^ value *p* value		χ^2^ value *p* value	
Province										
Alberta	319	10.2	144	10.1	265110	11.3	χ^2^ < 0.01 *p* = 0.92	h < 0.01	χ^2^ = 3.73 *p* = 0.05	h = -0.04
British Columbia	332	10.7	130	9.1	297400	12.7	χ^2^ = 2.38 *p* = 0.12	h = 0.05	χ^2^ = 11.93 *p* < 0.001	h = -0.07
Manitoba	120	3.8	53	3.7	89070	3.8	χ^2^ = 0.02 *p* = 0.90	h < 0.01	χ^2^ < 0.01 *p* = 0.95	h < 0.01
New Brunswick	90	2.9	36	2.5	49715	2.1	χ^2^ = 0.36 *p* = 0.55	h = 0.02	χ^2^ = 8.25 *p* < 0.01	h = 0.05
Newfoundland and Labrador	64	2.1	20	1.4	34210	1.5	χ^2^ = 1.95 *p* = 0.16	h = 0.05	χ^2^ = 7.07 *p* < 0.01	h = 0.05
Nova Scotia	138	4.4	50	3.5	61005	2.6	χ^2^ = 1.88 *p* = 0.17	h = 0.05	χ^2^ = 39.62 *p* < 0.001	h = 0.10
Ontario	926	29.7	400	28.0	938750	40.2	χ^2^ = 1.25 *p* = 0.26	h = 0.04	χ^2^ = 141.71 *p* < 0.001	h = -0.22
Prince Edward Island	26	0.8	7	0.5	10375	0.4	χ^2^ = 1.16 *p* = 0.28	h = 0.04	χ^2^ = 9.83 *p* < 0.01	h = 0.05
Quebec	1020	32.7	566	39.7	506640	21.7	χ^2^ = 20.44 *p* < 0.001	h = -0.14	χ^2^ = 222.49 *p* < 0.001	h = 0.25
Saskatchewan	82	2.6	21	1.5	74840	3.2	χ^2^ = 5.42 *p* = 0.02	h = 0.08	χ^2^ = 3.11 *p* = 0.08	h = -0.03
Language										
Bilingual	55	1.8	32	1.5	34560	2.2	χ^2^ = 0.95 *p* = 0.33	h = -0.03	χ^2^ = 1.55 *p* = 0.21	h = 0.02
English	1839	59	756	53	1238705	53.0	χ^2^ = 14.24 *p* < 0.001	h = 0.12	χ^2^ = 44.38 *p* < 0.001	h = 0.12
French	1030	33	560	39.2	432220	18.5	χ^2^ = 16.26 *p* < 0.001	h = -0.13	χ^2^ = 435.30 *p* < 0.001	h = 0.34
Other	191	6.1	78	5.5	630630	27.0	χ^2^ = 0.66 *p* = 0.42	h = 0.03	χ^2^ = 687.17 *p* < 0.001	h = -0.59
Gender										
Male	998	32.0	460	32.2	1075200	46.0	χ^2^ = 0.01 *p* = 0.91	h < -0.01	χ^2^ = 245.30 *p* < 0.001	h = -0.29
Female	2119	68.0	967	67.8	1260910	54.0	χ^2^ = 0.01 *p* = 0.91	h < 0.01	χ^2^ = 245.31 *p* < 0.001	h = 0.29
Education										
Elementary or High School	680	21.8	301	21.1	773935	33.1	χ^2^ = 0.26 *p* = 0.61	h = 0.02	χ^2^ = 179.37 *p* < 0.001	h = -0.25
College	1180	37.9	518	36.3	945980	40.5	χ^2^ = 0.95 *p* = 0.33	h = 0.03	χ^2^ = 8.87 *p* < 0.01	h = -0.05
University	1250	40.1	607	42.5	616200	26.4	χ^2^ = 2.30 *p* = 0.13	h = -0.05	χ^2^ = 301.14 *p* < 0.001	h = 0.29
Marital Status										
Single	228	7.3	107	7.5	109045	4.7	χ^2^ = 0.03 *p* = 0.87	h < -0.01	χ^2^ = 48.38 *p* < 0.001	h = 0.11
Married or Common Law	2545	81.6	1173	82.2	2030060	86.9	χ^2^ = 0.16 *p* = 0.68	h = -0.01	χ^2^ = 74.87 *p* < 0.001	h = -0.14
Separated/Divorced	339	10.9	145	10.2	197005	8.4	χ^2^ = 0.45 *p* = 0.50	h = 0.02	χ^2^ = 23.73 *p* < 0.001	h = 0.08
Employment Status										
Working full-time	2064	66.2	943	66.1	1245090	53.3	χ^2^ < 0.01 *p* = 0.96	h < 0.01	χ^2^ = 208.25 *p* < 0.001	h = 0.26
Working part-time	470	15.1	215	15.1	753585	32.3	χ^2^ = 0 *p* = 1	h < 0.01	χ^2^ = 419.79 *p* < 0.001	h = -0.41
Not working/Retired/Other	570	18.3	264	18.5	337445	14.4	χ^2^ = 0.02 *p* = 0.90	h < -0.01	χ^2^ = 36.86 *p* < 0.001	h = 0.10
Household Income (CAD$ before taxes)										
$39 999 or less	395	12.7	173	12.1	344940	14.8	χ^2^ = 0.22 *p* = 0.64	h = 0.02	χ^2^ = 10.67 *p* < 0.001	h = -0.06
between $40 000 and $59 999	428	13.7	187	13.1	332650	14.2	χ^2^ = 0.28 *p* = 0.60	h = 0.02	χ^2^ = 0.62 *p* = 0.43	h = -0.01
between $60 000 and $79 999	468	15.0	221	15.5	338000	14.5	χ^2^ = 0.14 *p* = 0.71	h = -0.01	χ^2^ = 0.71 *p* = 0.40	h = 0.02
between $80 000 and $99 999	511	16.4	237	16.6	323935	13.9	χ^2^ = 0.02 *p* = 0.89	h < -0.01	χ^2^ = 16.44 *p* < 0.001	h = 0.07
$100 000 or more	1009	32.4	459	32.2	996590	42.7	χ^2^ = 0.01 *p* = 0.92	h < 0.01	χ^2^ = 134.32 *p* < 0.001	h = -0.21
Nationality										
Born in Canada	2717	87.2	1263	88.5	1617475	69.2	χ^2^ = 1.50 *p* = 0.22	h = -0.04	χ^2^ = 469.17 *p* < 0.001	h = 0.44
Not born in Canada	397	12.7	164	11.5	718635	30.8	χ^2^ = 1.28 *p* = 0.26	h = 0.04	χ^2^ = 474.22 *p* < 0.001	h = -0.45
Ethnicity										
White (e.g., Caucasian, European)	2741	87.9	1280	89.7	1686435	72.2	χ^2^ = 2.81 *p* = 0.09	h = -0.06	χ^2^ = 383.89 *p* < 0.001	h = 0.40
East Asian (e.g., Chinese, Filipino, Japanese, Korean, Vietnamese)	119	3.8	49	3.4	203295	8.7	χ^2^ = 0.30 *p* = 0.58	h = 0.02	χ^2^ = 92.93 *p* < 0.001	h = 0.21
Other Ethnicities	231	7.4	84	5.9	446385	19.1	χ^2^ = 3.29 *p* = 0.07	h = 0.06	χ^2^ = 274.96 *p* < 0.001	h = -0.35
Religion										
Christian	1898	60.9	881	61.7	1578295	67.6	χ^2^ = 0.26 *p* = 0.61	h = -0.02	χ^2^ = 62.85 *p* < 0.001	h = -0.14
No Faith	984	31.6	444	31.1	485885	20.8	χ^2^ = 0.07 *p* = 0.79	h < 0.001	χ^2^ = 218.42 *p* < 0.001	h = 0.25
Other Faiths	170	5.5	74	5.2	271935	11.6	χ^2^ = 0.09 *p* = 0.76	h = 0.01	χ^2^ = 115.30 *p* < 0.001	h = -0.22

*StatCan* = Statistics Canada. *T1*:*T2* is the comparison between Time 1 sample and Time 2 sample. *T1:StatCan* is comparison between the Time 1 sample and Statistics Canada’s National Household Survey (2011) sample

### PAPM stage (1 item)

The primary outcome variable in our study was parents’ self-reported HPV vaccine decision-making stage, i.e., PAPM stage. Parents were asked: “Before today, which of the following best describes your thoughts about the HPV vaccine concerning my son?” Six response options were provided which allowed us to classify parents according to six distinct categorical stages of HPV vaccine decision-making (see Additional file 1). Of note, after assessing socio-demographics and HPV and HPV vaccine knowledge and just prior to assessing the outcome variable i.e., PAPM stage, participants were provided with a brief informative statement about HPV and the HPV vaccine in order to ensure that they had some basic awareness as to what HPV and the HPV vaccine was^4^.

### Psychosocial determinants

#### HPV and HPV vaccine knowledge items (36 items)

There is mixed evidence for the relationship between HPV-general and HPV-vaccine knowledge and parents’ HPV vaccination intentions/uptake for their child [40, 54, 55]. In order to assess what parents know about HPV and the HPV vaccine, we utilized Waller and colleagues existing psychometrically-tested, validated 16-item HPV and 7-item HPV vaccine scales [56]. We added 9 general HPV knowledge items and 4 HPV-vaccination specific items that were missing from the scale (e.g., items assessing about whether parents know about the link between HPV and other HPV-associated cancers beyond cervical cancer), (see Additional File 1). Items answered correctly were assigned 1 point while incorrect and “don’t know” were assigned 0 points to generate a total HPV-general and HPV vaccine knowledge scores.

#### Attitudes and beliefs (61 Items)

HPV-specific vaccination attitudes and general vaccine beliefs has been associated with parental vaccination intentions and uptake [37, 57]. The authors generated a list of 200 potential attitudinal items found after reviewing the psychosocial HPV vaccine literature and selected items based on constructs derived from different theoretical models of health behavior, including the HBM and the Theory of Reasoned Action) [58]. For each attitude and belief item, a 7-point Likert response format with 1 = Strongly Disagree, 4 = Neutral and 7 = Strongly Agree was used (see Additional file 1).

#### Information sources (6 items), health behaviors (6 items), implementation intentions (3 items) and other items

Participants were asked about the sources where they actually heard about the HPV vaccine and the sources they would prefer receiving information about the HPV vaccine. They were also asked if, and what type of recommendation (for, against, neutral, or neither) they received from a HCP for their son concerning the HPV vaccine. Self-reported health behaviors were also assessed e.g., whether their son has attended a routine medical check-up in the past year, acceptance of all the recommended childhood vaccines. Parents were asked who the primary health decision-maker was for their son (e.g., mother, father or joint).

Lastly, parents were also asked about behaviors they intended to complete at T1 (e.g., contact an HCP, search the internet), and at T2, using the computer-adaptive testing, we re-assessed if the specified behaviors they indicated at T1 were indeed carried out by T2.

Other additional items include: if the participant have any daughters and/or any vaccinated daughters (2 items); parent's health behaviours (3 items) communication about sex/HPV vaccination (7 items); degree of parental/son involvement in HPV vaccine decision-making (3 items), willingness to vaccinate at different price points (4 items); vaccine conspiracy beliefs (9 items) [59]; right wing authoritarianism (7 items); beliefs about other parents who do not vaccinate their child (2 items) and the Conspiracy Mentality Questionnaire (5 items) [60] are found in the additional file.

### Data cleaning

#### Addressing careless/unmotivated responders

Once data collection was completed, we sought to ensure the highest level of data quality and integrity of our conclusions. We used four data cleaning methods to identify participants who might not have used appropriate care while completing the questionnaire i.e., careless or unmotivated responders [61]. The four methods employed were: variance, bogus items, psychometric antonyms and psychometric synonyms [61]. For the variance criterion, we examined 64 items (some reverse coded) dispersed across 7 separate web pages in our online questionnaire. There were 13, 9, 7, 11, 8, 10 and 6 items across the 7 different web pages. We flagged participants who had 0 variance across the items on more than 4 of the 7 pages.

For the validity criterion, we used three bogus items from Weinberger and colleagues [62]: “I have never met anyone younger than I am”; “Everyone makes mistakes at least once in a while”, and “I am answering these questions truthfully” with response options ranging from 1 = “strongly disagree” to 7 = “strongly agree”, where 4 = “neutral”. We reverse coded the first item and created a total validity score for the three items. We removed participants who scored 12 or below, then, re-introduced any participant who answered “neutral” to all three items. The rationale for this cut-off was that we sought to identify participants who scored below “neutral” (somewhat disagree, disagree and strongly disagree) given that the correct answer to these items was to “agree” with them (note that the opposite is true for the one reverse coded item). We chose to re-introduce any participant who answered “neutral” to all three items as these items are available for subjective interpretation and those who were systematically answering “neutral” to *all* items would be removed by the variance method.

Another method used was psychometric synonyms and antonyms [61, 63], which are consistency indices that help to eliminate bias by examining differences in items that are highly similar or opposing in content. We examined any questionnaire item that had a 7-point Likert response option. Post hoc, we standardized all relevant items into *z* scores and correlated all items. We identified the 30 most positively and 30 most negatively correlated items. We recoded these items to create pairs of synonyms and then calculated the correlation between synonyms and antonyms for each participant, which established a synonym index and an antonym index for each participant. We then flagged all values less than -2 standard deviations (*SD*) on the synonyms index and greater than +2 *SD* on the antonyms index as these correlations could be seen as extreme outliers.

These four methods identified that 15% of our sample at T1 (*n* = 575) and 5% of our sample (*n* = 202) at T2 belonged to a latent class that can be considered careless or unmotivated in their responses, a percentage nearly identical to findings by other research groups [61, 64]. Data collected from these participants were removed from our final sample (see Fig. 1).

### Self-report of son’s vaccination status

Following T1 data collection, we inspected the data from our primary outcome variable, PAPM stage. The authors observed that some participants’ responses were implausible, nonsensical or inaccurate. We speculated that perhaps parents may have confused the HPV vaccine with other childhood vaccinations, and therefore some participants likely did not match the profile of a participant who had truly vaccinated his or her son against HPV. For example, some participants indicated that their son had been vaccinated in school, even though they lived in provinces where indeed no school-based programs for boys yet existed. This challenge of *self-report* (i.e., subjective) vaccination as opposed to *objective* (e.g., vaccination booklet with official stamps or electronic vaccination registries) has been reported in the literature [65, 66].

During data cleaning at T1, the authors therefore established a first method of examining a set of 10 criteria to increase the likelihood that parents who had indicated that they had vaccinated their son were not false positives. Furthermore, in order to improve upon the accuracy of parents’ self-reported vaccination status, at T2, we prompted those who selected PAPM stage 6 (i.e., vaccination) with a brief informative statement about the Canadian HPV vaccine policy (e.g., we informed participants that except for PEI, parents have to pay/purchase the HPV vaccine) and then asked the PAPM stage item a second time to ensure that their PAPM stage was as accurate as possible.

At T2, two additional issues arose. The first issue was impossible PAPM stage transitions. From both a theoretical and practical perspective, it is impossible for someone to report being in stages 2–6 at T1, and then to report being in stage 1 at T2. For such a report to be true, the participant would need to have become *unaware*, after having previously been *aware* that the HPV vaccine could be administered to males. The second issue was the impossibility of someone moving from reporting that their son had been *vaccinated* (Stage 6) at T1, to then reporting any other stage at T2 (i.e. implying that their son is no longer vaccinated). In total, using the aforementioned three methods, 92 participants were removed from the final sample due to likely inaccurate or implausible vaccination stage (see Fig. 1).

Statistical analysis was conducted using SPSS v23 and R v3.2.2.

## Results

The mean time to complete the questionnaire was 21 min at T1 and 24 min at T2.

### Participants and socio-demographics

The final cohort consisted of 3,117 participants at T1 and 1,427 at T2, representing a 45% attrition rate (see Fig. 1 for recruitment overview). The response rate, calculated based on completion by participants who initiated the questionnaire (*n* = 5733 at T1 and *n* = 1999 at T2), was 66.0% at T1 and 80.4% at T2. The sample’s socio-demographic characteristics are presented in Table 1.

When comparing the T1 and T2 samples, the samples were found to be similar as there were no significant differences on all socio-demographic variables except for two provinces and language (see Table 1). We had significantly more respondents from Quebec and fewer respondents from Saskatchewan at T2 compared to T1 but the difference was small (h ≤ 0.2). We also had fewer English respondents and more French respondents at T2 compared to T1, and the difference was small as well (h ≤ 0.2).

A comparison of the T1 and Statistics Canada samples revealed that there were statistically significant differences for the proportions of responses between the two samples for all provinces (except Alberta, Manitoba and Saskatchewan), language (except bilinguals), gender, education, marital status, employment status, income (except those earning between $40 000 and $59 999 and those earning between $60 000 and $79 999), nationality, ethnicity and religion (see Table 1). An examination of the effect size indicates that the effect size was small for 14 differences and medium for 18 differences (see Table 1). In no case, was the effect size large (see Table 1).

### PAPM stages

The number and percentage of parents across the six PAPM stages is presented in Table 2. The HPV vaccination uptake of Canadian males 9-16 years old was very low, with only 34 and 39 parents reporting that their sons were vaccinated at T1 and T2, which represents an HPV vaccine uptake rate of 1.1% at T1 and 2.7% at T2. Of the few parents who indicated that they had vaccinated their son, 47% received one dose at T1 and 56% at T2 (*p* > 0.05). Two or three doses were reportedly received by 53% of sons at T1 and 44% at T2 (χ^2^ = 0.32, CI: -0.34; 0.16, *p* > 0.05).

**Table 2:** PAPM stages at Time 1 and Time 2

PAPM Stage	Time 1		Time 2	
	*n*	%	*n*	%
I was *unaware* that the HPV vaccine could be given to males (Stage 1)	1778	57.0	218	15.3
I was aware that the HPV vaccine can be given to males, but I have not thought about getting the HPV vaccine for *my son* (*unengaged*, Stage 2)	652	20.9	462	32.4
I have thought about getting the HPV vaccine for *my son*, but I am *undecided* about getting the HPV vaccine for him (Stage 3)	284	9.1	360	25.2
I have decided I do NOT want *my son* to get the HPV vaccine *(Stage 4, decided not to*)	212	6.8	208	14.6
I have decided I DO want *my son* to get the HPV vaccine (Stage5*, decided to*)	157	5.0	140	9.8
*My son* has already received the HPV vaccine (Stage 6, vaccinated)	34	1.1	39	2.7

While there was a free school-based program in place for boys in Grade 6 in PEI, our results still show that 19 parents from PEI were unaware, unengaged or undecided. At T2, there was a program in place for boys in Grade 5 in Alberta, and our results indicate that 85 parents were unaware, unengaged or undecided from these two provinces. Moreover, at T1, only 1 parent from the 34 (2.9%) who reported their son was vaccinated were from provinces offering free HPV vaccination for boys (PEI) and at T2, 11 from the 39 (28.2%) were parents of vaccinated sons who were from provinces that were vaccinating boys against HPV as part of the provincial immunization schedule (PEI and Alberta).

### HPV and HPV vaccine knowledge

We validated and extended Waller et al.’s existing knowledge scales and create a 25-item HPV general knowledge scale and the 11-item HPV vaccine knowledge scale, which were found to be psychometrically robust [67].

The mean scores for HPV knowledge were 11.67 (from 25 possible points) at T1 and 14.02 at T2 (t = 12.11, CI: 1.97; 2.73, *p* < 0.01). The mean scores for HPV vaccination knowledge were 5.22 (from 11 possible points) at T1 and 6.3 at T2 (t = 12.27, CI: 0.9; 1.24, *p* < 0.01).

A detailed elaboration of these results (e.g., what parents know/don’t know) and the relationship between knowledge and PAPM stages are presented elsewhere [67].

### Attitudes and beliefs

We developed and validated a comprehensive, psychometrically-sound HPV vaccination attitudes and belief scale (HABS), which contains 46 items and 9 factors: benefits, threat, influence, harms, risk, affordability, communication, accessibility and general attitudes (see Additional file 1). The psychometric properties of the scale are described in another paper [68].

### Information sources

Most parents (94% at T1 and 88% at T2) never spoke with a doctor /HCP about the HPV vaccine for their son (χ^2^ = 40.4, CI: 0.04; 0.08 *p* < 0.01, h = 0.2).

**Fig. 2:**
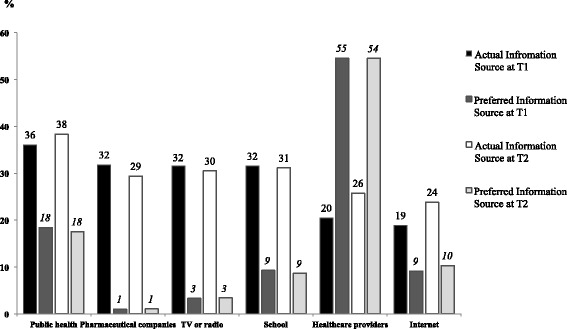
Percentage of participant’s actual source of receiving HPV vaccine information compared to their preferred information sources at both Time 1 and Time 2

Of the few parents (6% at T1 and 11.4% at T2) who did speak to their doctors/HCP, 59% of them were recommended to get the HPV vaccine for their son at T1 and 69% at T2 (χ^2^ = 3.33, CI: -0.21; 0.006, *p* = 0.07). At T1, 55.8% of those parents who vaccinated their son had spoken with a HCP about the HPV vaccine. More than half the sample (54%) at both T1 and T2 prefer to receive their information from an HCP, which was by far the most preferred source of information, followed by public health brochures, pamphlets, flyers or posters which was reported by 18% of parents at T1 and T2. Parents reported that the sources from which they actually received information about the HPV vaccine (e.g., TV or radio) did not correspond with their most preferred information source (e.g., from their HCP, see Fig. 2).

### Health behaviors

At both time points, parents indicated that their son’s healthcare decisions are typically a joint decision made by both parents (60.4% at T1 and 62% at T2, χ^2^ = 0.5, *p* > 0.05), followed by mothers alone (40.2% at T1 and 39% at T2, χ^2^ = 1.05, *p* > 0.05) and fathers alone (5% at T1 and 4.6% at T2, χ^2^ = 0.28 *p* > 0.05).

More than half of parents (61% at T1 and 59% at T2) mentioned that their son underwent a routine checkup with a healthcare provider in the previous year (χ^2^ = 2.07, CI: **-**0.008; 0.054, *p* > 0.05). Most parents (93% at both T1 and T2) stated that their sons have received all childhood vaccines. Interestingly, at T1 and T2 respectively, 25.5% and 21.2% of parents who decided not to vaccinate their son against HPV stated their son did not receive all recommended childhood vaccines; the proportions were significantly higher than parents belonging to any of the other 5 PAPM stages at both time-points (*p* < 0.05).

### Implementation intentions

**Fig. 3:**
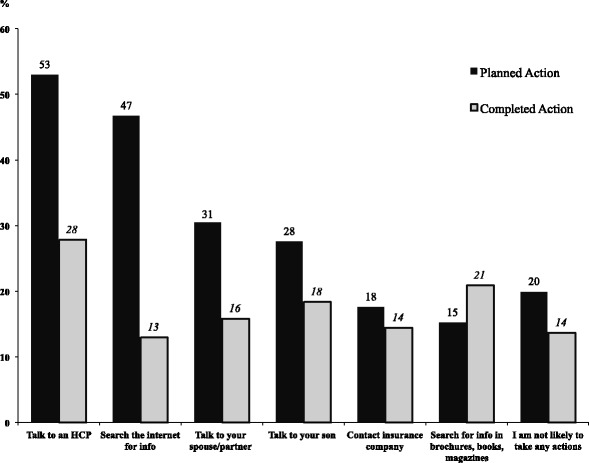
Percentage of participant’s self-reported planned actions at Time 1 compared to actions reported as completed at Time 2 (Implementation Intentions)

In most cases, parents did not implement their planned/intended actions to facilitate HPV vaccination between T1 and T2. Parents increased the search for information about HPV vaccine in written sources (i.e., brochures, books, magazines) at T2 (21%) compared to T1 (15%) (see Fig. 3). Some parents did not name a planned intention, but when they stated nothing, they indeed remained in status quo.

### Stage transitions from T1 to T2 (*n* = 1427)

**Fig. 4:**
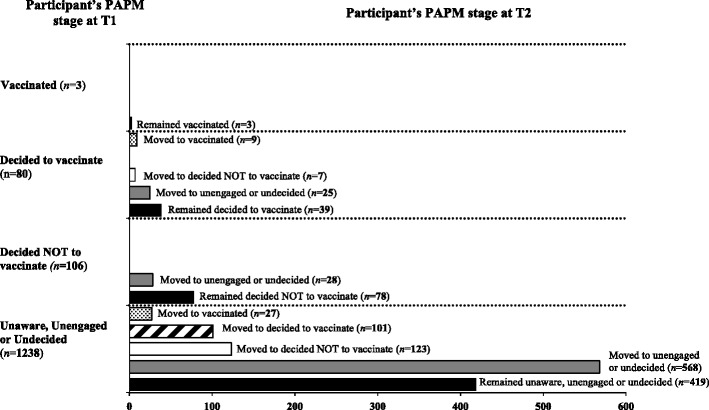
Number of participant’s initial PAPM stage reported at T1 is shown on the y-axis. Number of participant’s who remained in the same PAPM stage or their movement to a different PAPM stage at T2 shown on the x-axis (*n* =1427)

We had 539 (37.7%) participants who remained in the same stages of vaccination adoption (i.e., PAPM stage) from T1 to T2; this includes 3 participants who indicated at T1 that their sons were vaccinated. A higher number, 705 (49.4%) progressed from T1 to T2 towards advanced PAPM stages that are closer to action i.e., vaccination; 53 participants (3.7%) regressed (to earlier stages than they initially were in, away from action); only 36 parents (2.5%) advanced to having their sons vaccinated at T2. Of the 1238 participants who initially identified as being unaware, unengaged or undecided at T1 and who completed the T2 questionnaire, 27 progressed to vaccinated at T2. Of the 80 participants who had decided to act at T1 and who completed the T2 questionnaire, only 9 participants (11%) progressed to being vaccinated at T2. Not a single participant in stage 4 in T1 (i.e. decided not to act, *n* = 106) moved towards decided to act or vaccination at T2. 130 participants (9.1%) moved from unaware, unengaged, undecided or decided to act at T1 to decided not to act at T2 (see Fig. 4).

## Discussion

To our knowledge, this is the first HPV vaccination specific survey in a pan-Canadian representative sample of parents of boys after the first HPV vaccine (Gardasil®) was licensed in Canada for males in September 2010 [14]. Other vaccination surveys such as the Childhood National Immunization Coverage Survey (CNICS) conducted by Statistics Canada have not been gender and HPV specific [69], such that the data collected are less representative of the Canadian population of parents of boys and do include items about HPV vaccination for males. At the time of data collection, only one of the ten provinces at T1 (PEI), and two of the provinces at T2 (PEI and Alberta) had implemented school-based HPV vaccination program for males, and no territories offered school-based HPV vaccination for boys. As such, only a small number of parents from PEI and Alberta and (i.e., only those with sons in grade 6 and 5 respectively) were eligible for free school-based HPV vaccination programs. In the absence of programs, the HPV vaccine uptake, was exceptionally low at both T1 (1.1%, *n* = 34 from 3117) and T2 (2.7%, *n* = 39 from 1427).

Similarly, the lack of programs for boys, and in turn the cost of vaccinations as well the lack of information (e.g., not even knowing boys can get the HPV vaccine; lack of understanding about the benefits/risks; no recommendation from a HCP) likely explains why at both time-points most parents (87% at T1 and 73% at T2) were in the first three stages of adoption (unaware, unengaged or undecided). Furthermore, post-hoc, we examined the few sons (*n* = 34 and T1 and *n* = 39 at T2) who were vaccinated, and the majority was not *even* from provinces that offered free-school based HPV vaccination programs. Having two provinces that had introduced male HPV vaccination programs did not appear to skew our ‘snapshot’ of parental HPV vaccine decision. We were also able to establish a reliable estimate of HPV vaccine uptake in Canada. Currently (as of September 2016), there are six Canadian provinces with HPV vaccination programs for males. The six Canadian provinces join only a handful of other countries/regions e.g., Australia, Austria, Israel, Barbados, Lichtenstein, New Zealand that have implemented or are set to implement publicly funded HPV vaccination programs for boys [25, 29, 30]. Our work offers valuable baseline information to all stakeholders involved in implementing and evaluating HPV vaccination programs.

At T2, almost half the sample moved forward along the PAPM vaccine decision-making trajectory, with most moving towards unengaged or undecided. Over a third of the sample remained in the same stage as at baseline. These results are not surprising, considering our study was an observation design and not an intervention study. Moreover, the movement towards the later stages of adoption was minimal, i.e., very few parents moved towards deciding to act and acting/vaccination. In the absence of programs or targeted interventions that match parents’ informational needs, most parents remained fixed in their current and/or earlier stages of adoption. The forward movement along the PAPM vaccine decision-making trajectory could likely be explained by parents acquiring information through written sources (e.g., media) or simply by virtue of completing the questionnaire at T1. Furthermore, voluntary initiation of parents e.g. to acquire information via the internet or to speak to their HCP was not found at T2. Of those parents who had decided to vaccinate their son at T1 i.e., had intentions and who completed the T2 questionnaire, very few parents followed through in vaccinating their sons even when they were in the later stages of decision-making. This finding supports a growing body of research showing that there are important gaps between intending to act and carrying out intentions [70]. Therefore, some individuals likely require help developing specific implementation plans to reduce the barriers.

Of interest, the most immobile group were those who had decided not to vaccinate, with no parent in this stage (of 106) moving toward intentions or vaccination at T2. Our results complement previous research suggesting that a proportion of these parents may likely be hesitant towards *all* vaccines and not uniquely against the HPV vaccine, and perhaps more akin to what are known as “anti-vaxxers” [71, 72].

For the entire sample, HPV knowledge and HPV vaccine knowledge remained poor at both time-points. The relationship between parent’s knowledge and vaccine acceptance/intentions is mixed and equivocal [73–77]. Low knowledge in the present group of parents could be explained by the relatively new recommendation of the HPV vaccine for boys and indicate the need to inform parents about the link between HPV and penile, anal and oral cancers as well as GW. Importantly, there were discrepancies between preferred and actual HPV information channels. Although parents are requesting and requiring more information on HPV vaccination, their needs are not being met. Providing relevant, accurate information about the recommendation and benefits of the HPV vaccine for boys, ideally delivered by a doctor or HCP, could improve HPV vaccine uptake.

Our results also indicated that the vast majority of Canadian parents have not received a recommendation from their HCP about the HPV vaccine for their sons despite their HCP being the primary source they prefer and want to receive information from. Moreover, while the sample size is small (*n* =36), 80% of parents who advanced towards actual vaccination from T1 to T2 received a recommendation from their HCP. An HCP recommendation has almost consistently been shown to be associated with increased parental HPV vaccine acceptability [73, 76, 78–80] and the absence of an HCP recommendation has been associated with negative attitudes and refusal of HPV vaccination [74–76, 80]. Facilitating knowledge translation through HCPs should be a major goal for future interventions to increase HPV knowledge and in turn, improve HPV vaccination uptake [14]. Other potential avenues where parents could acquire HPV information is from public health brochures, pamphlets and posters provided by government health organizations and endorsed by different medical organizations (e.g. Canadian Medical Association) which may be seen as an HCP endorsement. Since other vaccines (e.g., Tdap, Hepatitis B and meningococcal) are given to Canadian children at a similar age/grade as the HPV vaccine, an opportunity exists to pair the vaccines together in administration and educate parents about HPV vaccination.

The present study’s strengths are related to the study’s longitudinal design, data collection tool (questionnaire), data collection method (online survey to acquire a pan-Canadian sample) and data cleaning techniques. The online survey approach allowed us to: 1) use computer-adaptive testing, 2) avoid missing data, and 3) collect data in a time efficient way. Furthermore, by developing a strong data-cleaning algorithm, we increased the reliability of our final data. Moreover, the authors engaged in extensive psychometric testing [67, 68] to ensure the validity of the psychosocial constructs which has been recommended in this area of research [33]. Additionally, our study utilized a longitudinal design, which will allow us to analyze how the psychosocial determinants influence HPV vaccine decision making over time. To the best of our knowledge, there is only one existing longitudinal study of parents of boys which was conducted outside of Canada [81]. Moreover, our results confirmed that intentions do not translate into vaccination over time (only 7/80 of the decided to when on to vaccinate their sons), which is often unknown in most intention studies. Lastly, the use of the PAPM allowed us to capture HPV vaccine decision-making in a more nuanced way, and not presume that all parents are aware or engaged in this particular health behavior. Therefore, our results demonstrate that in studying HPV decision-making, the PAPM is likely a fitting theoretical model in contrast to the HBM or IBM, which ignore the earlier stages of vaccine decision-making.

Our study is limited in several ways. Compared to data collected from Statistics Canada household survey sample of parents with 9-16-year-old, there were differences in the structure of our sample. The effect size was mostly small to medium with no effect size exceeding 0.6. In our opinion, the small to medium differences allow us to generalize our results to the Canadian context. Our suggestion for future studies would be to impose quotas based on the repartition of respondents consistently with national representative available data in order to further reduce sample differences. Additionally, our sample consisted of more mothers (65%) than fathers (35%). Importantly, our response rate of males is higher than in other studies reporting HPV vaccine related attitudes where the average proportion of mothers was 82.3% [37]. Therefore, in our opinion the proportion of males and females in our sample closely reflects the gender specific HPV vaccination decision-making process in Canada. Participants were also lost to follow-up, but importantly our T2 sample was comparable and nearly identical to the original T1 sample on all socio-demographic variables albeit province and language, where the effect was small. Moreover, we were unable to sample the three Northern territories constituting of mostly Indigenous peoples (e.g., North American Indian, Inuit), as these residents were not represented in Leger’s panel. Future research should evaluate the psychosocial determinants of HPV vaccine decision-making in this population. Additionally, the present findings did not analyze the confounding role of having daughters who are eligible for HPV vaccination in the household. Future analyses are underway which will examine whether having a vaccinated daughter is a predictor of PAPM stage. A final limitation was the recall bias of some participants’ self-reported vaccination status. The issue of inaccurate *self-report* of vaccination as opposed to *actual* (e.g. vaccination booklet, physician’s record) has been reported in the vaccine literature [65]. In the absence of an HPV immunization school-based program, most males receive the HPV vaccine privately and the option to register this information with national databases is voluntary. As the HPV vaccination rate was extremely low (1-3%) in our study, and the HPV vaccine was not included in provincial immunization programs, we have reason to believe that the lack of objective proof of vaccination had only a small influence on our results. Future studies should consider that parents’ self-reported vaccinated status may be unreliable and should try to use objective records to precisely measure HPV vaccine uptake.

## Conclusions

Our results illustrate the exceptionally low uptake of the HPV vaccine in Canadian boys in the absence of a funded immunization program. Parents are critical to a successful HPV vaccination program in children. Directing our attention to males as much as females is important because males play a role in transmission and are vulnerable to HPV-associated diseases. This data can help direct efforts towards helping Canadians become aware that males are recommended to get the HPV vaccine and be engaged in the decision to vaccinate their sons.

Moreover, intentions to vaccinate one’s son or planning to speak to one’s HCP did not translate into action for most parents over the 9-month follow-up. These results have implications for implementation of strategies (e.g., HCPs offering the HPV vaccine to the parent of a son directly and immediately during a routine visit, fostering resources within schools to increase HPV vaccine uptake). Lastly, the use of staged-based health behavior model, i.e., the PAPM, allowed for more precision as to where parents stood along the HPV vaccine decision-making trajectory. Forthcoming analyses to better understand the psychosocial determinants that influence each specific stage will allow us to target the unique gaps and barriers of each PAPM stage.

## Additional file

Additional file 1: Questionnaire Sections with variable constructs, number of items, sample items and response choices. (DOCX 142 kb)

## Abbreviations

BC: British Columbia
CNICS: Childhood National immunization coverage survey
GW: Genital warts
HABS: HPV Attitudes and Beliefs Scale
HBM: Health belief model
HCP: Healthcare provider
HPV: Human papillomavirus
IBM: Integrated behavioral model
MSM: Men having sex with men
NACI: Canada’s National advisory committee on immunization
PAPM: Precaution adoption process model
PEI: Prince Edward Island

## Footnotes

1 The PAPM original model consist of seven stages of health decision-making. The seventh stage is a maintenance stage and does not apply to HPV vaccination. For simplicity, only the six stages are described.

2 Leger has the largest representative panel in Canada and the largest Franco-Canadian panel. Since members are recruited randomly over the phone, the Leger Panel is highly representative and offers exceptional quality respondents. Leger sampling process is based on data from Statistics Canada (e.g., province, age, gender, language and region).

3 As the questionnaire was computer adapted, few items were asked of only some groups. For example, only those participants who indicated they vaccinated their son, were further asked about how many doses he received.

4 The informative statement read as follows: Please read carefully the following information about HPV. The Human Papillomavirus (HPV) is the most common sexually transmitted infection. HPV can cause genital warts. HPV can also cause cancers of the cervix, penis, anus, vagina, vulva and oral cancers. There are HPV vaccines available that are sometimes referred to as the cervical cancer vaccine, Gardasil®, or Cervarix®. The HPV vaccine is given in 2 or 3 doses and costs approximately $150–$200 per dose. Health Canada has approved and recommended an HPV vaccine for both males aged 9–26 years and females aged 9–45 years.
