# Supplementary material for: Psychosocial determinants of parental human papillomavirus (HPV) vaccine decision-making for sons: Methodological challenges and initial results of a pan-Canadian longitudinal study
Source: BMC Public Health. 2016 Dec 5;16:1223. doi: 10.1186/s12889-016-3828-9 (PMC5139028; doi:10.1186/s12889-016-3828-9)
Supplement: Additional file 1: — Questionnaire Sections with variable constructs, number of items, sample items and response choices. (DOCX 142 kb) [file 12889_2016_3828_MOESM1_ESM.docx]

| **Variable Construct** | **# of Items** | **Sample Items** | **Response Choices** |
| --- | --- | --- | --- |
| **Socio-Demographics** (e.g., Province, Sex, Parent’s Age, Language, Education, Marital Status, Employment Status, Urbanity, Canadian born, Years in Canada, Ethnicity, Religion, Income, Son's age) | 12 | What is the language you first learned at home in your childhood and that you still understand? With which religious or spiritual belief system do you most strongly identify? | Categories derived from those commonly used by Statistics Canada. Prefer not to answer was an option for some items. |
| **Religiosity** | 1 | Please rate how much you agree with the following statement: My religious or spiritual belief system guides my daily decisions. | Strongly Disagree – Strongly Agree (7-point Likert scale) |
| **HPV & HPV Vaccine Awareness** | 2 | Have you ever heard of HPV (Human Papillomavirus)? Have you ever heard of the HPV vaccine (Human Papillomavirus vaccine)? You may also have heard of this vaccine under the names Gardasil® or Cervarix®. | Yes-No |
| **HPV & HPV Vaccine Perceived Knowledge** | 2 | How much would you say you know about HPV (Human Papillomavirus)?  How much would you say you know about the HPV vaccine (Human Papillomavirus vaccine, also referred to as Gardasil® or Cervarix®)? | Nothing at all – A lot (4-point Likert scale) |
| **Precaution Adoption Process Model** | 1 | Before today, which of the following best described your thoughts about the HPV vaccine concerning *my son^[[1]](#footnote-1)^*? | **Stage 1:** I was unaware that the HPV vaccine could be given to males **Stage 2:** I was aware that the HPV vaccine can be given to males, but I have not thought about getting the HPV vaccine for *my son* **Stage 3:** I have thought about getting the HPV vaccine for *my son*, but I am undecided about getting the HPV vaccine for him **Stage 4:** I have decided I do NOT want my son to get the HPV vaccine **Stage 5:** I have decided I DO want *my son* to get the HPV vaccine **Stage 6:** *My son* has already received the HPV vaccine |

| **Variable Construct** | **# of Items** | **Sample Items** | **Response Choices** |
| --- | --- | --- | --- |
| **HPV Knowledge^[[2]](#footnote-2)^** | 25 | HPV is very rare (F) HPV can cause genital warts (T) | True; False; Don't know |
| **HPV Vaccine Knowledge2** | 11 | The HPV vaccines offer protection against all sexually transmitted infections (F) The HPV vaccines are most effective if given to people who've never had sex (T) | True; False; Don't know |
| **Attitudes and Beliefs ^[[3]](#footnote-3)^** | 61 | **Benefits (10):** I feel that the HPV vaccine is effective in preventing genital warts  **Threat (3):** I feel that it would be serious if m*y son* contracted HPV-related cancer later in life.  **Influence (8):** I feel that other parents in my community are getting their sons the HPV vaccine.  **Harms (6):** I feel that the HPV vaccine may lead to long-term health problems  **Risk (3):** I feel that without the HPV vaccine, *my son* would be at risk of getting HPV-related cancer later in life  **Affordability (3):** I feel that the HPV vaccine is too expensive  **Communication (5):** I feel that it is hard to talk to *my son* about his sexual health  **Accessibility (4):** I feel that dealing with getting the HPV vaccine for *my son* would be simple  **General Vaccinations Attitudes (4):** I do not like the idea of vaccines . | Strongly Disagree – Strongly Agree (7-point Likert scale) |
| **Information Sources** | 6 | Where have you heard about the HPV vaccine in general (other than this survey)?  From which sources would you prefer to receive information about the HPV vaccine? Which is your most preferred source? | E.g. Public health brochures, doctor, nurse, or other health care provider, school, internet, etc |

| **Variable Construct** | **# of Items** | **Sample Items** | **Response Choices** |
| --- | --- | --- | --- |
| **Doctor Discussion** | 1 | Have you ever talked with a doctor/health care provider about the HPV vaccine for *my son?* | No; Yes, and he/she recommended that *my son* get the HPV vaccine; Yes, and he/she has no opinion about the HPV vaccine for *my son*; Yes and he/she recommended against *my son* getting the HPV vaccine; Yes, but he/she recommended to wait until he’s older before giving *my son* the HPV vaccine; Other, please specify. |
| **Son’s Health Behaviours** | 6 | Who normally makes *my son* healthcare decisions?: Has *my son* gone for a routine medical check-up with a doctor/health care provider in the last year? Has *my son* received all the recommended childhood vaccines? | E.g. Mother/female guardian; Joint decision between parents/guardians  Yes; No; I don’t know |
| **Parent Heath Behaviours** | 3 | Have you ever been told that you have a sexually transmitted infection or disease (e.g., HPV, chlamydia, genital herpes, syphilis, etc.)?  Have you ever been diagnosed with cancer? | Yes; No; I prefer not to answer |
| **Vaccinated Daughters** | 4 | How many daughters do you have?  How many of your daughters have received the HPV vaccine | Specify numbers |
| **Communication about Sex and HPV vaccine** | 7 | How much have you talked with *my son* about sex? Has *my son* ever mentioned to you that he would like to get the HPV vaccine? When you talked to friends, peers or co-workers about the HPV vaccine, this was about: | Not at all – A lot (4-point Likert scale) Yes; No E.g. Sex and other topics of a sexual nature; Risks and side effects of the HPV vaccine |
| **Parent/Son Involvement** | 3 | How involved do you feel you should be in the decision to get *my son* the HPV vaccine? How involved do you feel your son’s other parent should be in the decision to get *my son* the HPV vaccine? | Not at all involved – Extremely Involved (5-point Likert scale) |
| **Willingness to vaccinate at different price points** | 4 | Please indicate how willing you would be to get all the HPV vaccine doses for *my son* if vaccinating *my son* against HPV would cost $300? (from your own money, without any insurance or government coverage) | Extremely unwilling – Extremely willing (5-point Likert scale) |

| **Variable Construct** | **# of Items** | **Sample Items** | **Response Choices** |
| --- | --- | --- | --- |
| **Implementation Intentions** | 3 | You indicated that you decided you DO want *my son* to get the HPV vaccine. Which of the following best describes your thoughts?^[[4]](#footnote-4)^ I have taken the following actions since deciding that *my son* will get the HPV vaccine:^4^  Now that you have completed this survey, which of the following are you likely to do? **^[[5]](#footnote-5)^** | E.g. I plan on getting *my son* his first HPV vaccine dose within the next 6-12 months; I do not know when I plan on getting *my son* the HPV vaccine E.g. I contacted a health care provider to ask questions; I set aside money to pay for the HPV vaccine  E.g. I am not likely to take any actions; Search for information about HPV and/or the HPV vaccine on the internet; Contact your insurance company to see if they cover any of the costs of the HPV vaccine; Talk to your doctors/health care provider about HPV and/or the HPV vaccine; |
| **Open Ended Qualitative Items** | 4 | What would influence your decision to have *my son* vaccinated or not against HPV?  What do you remember hearing in the media about the HPV vaccine?  What questions do you need answered to make a decision regarding the HPV vaccine for your son*?* | Free-text responses |
| **Vaccine Conspiracy Beliefs^[[6]](#footnote-6),^^[[7]](#footnote-7)^** | 9 | Immunizing children is harmful and this fact is covered up | Strongly Disagree – Strongly Agree (7-point Likert scale) |
| **Right Wing Authoritarianism^6^** | 7 | Everyone should have their own lifestyle, religious beliefs, and sexual preferences, even if it makes them different from everyone else | Strongly Disagree – Strongly Agree (7-point Likert scale) |
| **Beliefs about other parents vaccination choices^6^** | 2 | Parents who don’t vaccinate their children with the HPV vaccine are putting *my child* at risk  Parents who don’t vaccinate their children with the HPV vaccine are putting *their child* at risk | Strongly Disagree – Strongly Agree (7-point Likert scale) |
| **Conspiracy Mentality Questionnaire^6,^^[[8]](#footnote-8)^** | 5 | I think that many very important things happen in the world, which the public is never informed about | Certainly Not (0%)– Certain (100% (11-point Likert scale) |

1. Participants were asked at the beginning of the questionnaire to provide a name, nickname, initials or abbreviations for their son who is between the ages of 9 and 16 and who has had the nearest birthday. Using intelligence programming, Parents’ sons initials, name, nickname (e.g., JT, Dan) was then replaced for “*my son*” in all items, making the questionnaire individualized for each participant. [↑](#footnote-ref-1)
2. Waller J, Ostini R, Marlow LA, McCaffery K, Zimet G. Validation of a measure of knowledge about human papillomavirus (HPV) using item response theory and classical test theory. Preventive Medicine. 2013; 56(1):35-40. doi: 10.1016/j.ypmed.2012.10.028

   2Perez S, Tatar O, Ostini R, Shapiro GK, Waller J, Zimet G, et al. Extending and validating a human papillomavirus (HPV) knowledge measure in a national sample of Canadian parents of boys. Preventive Medicine. 2016; 91:43-9. doi: 10.1016/j.ypmed.2016.07.017 [↑](#footnote-ref-2)
3. Perez S, Shapiro GK, Tatar O, Joyal-Desmarais K, Rosberger Z. Development and Validation of the Human Papillomavirus Attitudes and Beliefs Scale in a National Canadian Sample. Sexually Transmitted Diseases. 2016:1. doi: 10.1097/olq.0000000000000506 [↑](#footnote-ref-3)
4. Asked to participants in Stage 5 only [↑](#footnote-ref-4)
5. The actual behaviours that they selected at T1 were shown at T2 to assess if the actions they planned were indeed carry out: You indicated in February that you were planning the following actions. Which one(s) have you done since then? [↑](#footnote-ref-5)
6. These items were only asked to participants at T2 at the end end of the entire questionnaire. [↑](#footnote-ref-6)
7. Shapiro GK, Holding A, Perez S, Amsel R, Rosberger Z. 2016. Validation of the Conspiracy Beliefs Scale. Papillomavirus Research, 2, 167-172. doi: 10.1016/j.pvr.2016.09.001 [↑](#footnote-ref-7)
8. Bruder M, Haffke P, Neave N, Nouripanah N, Imhoff R. Measuring individual differences in generic beliefs in conspiracy theories across cultures: conspiracy mentality questionnaire. Front Psychol. 2013;4:225. doi: 10.3389/fpsyg.2013.00225 [↑](#footnote-ref-8)
